# Theoretical analysis of immunochromatographic assay and consideration of its operating parameters for efficient designing of high-sensitivity cardiac troponin I (hs-cTnI) detection

**DOI:** 10.1038/s41598-023-45050-1

**Published:** 2023-10-25

**Authors:** Rahul Agarwal, Sergio Omar Martinez-Chapa, Marc Jozef Madou

**Affiliations:** 1 School of Engineering and Sciences, Tecnológico de Monterrey, Ave. Eugenio Garza Sada 2501, 64849 Monterrey, NL Mexico; 2 Autonomous Medical Devices Incorporated (AMDI), 3511 W Sunflower Ave, Santa Ana, CA 92704 USA; 3 Mechanical and Aerospace Engineering, University of California, Irvine, USA

**Keywords:** Laboratory techniques and procedures, Biomedical engineering, Fluid dynamics

## Abstract

Troponin is the American College of Cardiology and American Heart Association preferred biomarker for diagnosing acute myocardial infarction (MI). We provide a modeling framework for high sensitivity cardiac Troponin I (hs-cTnI) detection in chromatographic immunoassays (flow displacement mode) with an analytical limit of detection, i.e., LOD < 10 ng/L. We show that each of the various control parameters exert a significant influence over the design requirements to reach the desired LOD. Additionally, the design implications in a multiplexed fluidic network, as in the case of Simple Plex™ Ella instrument, are significantly affected by the choice of the number of channels or partitions in the network. We also provide an upgrade on the existing LOD equation to evaluate the necessary minimum volume to detect a particular concentration by considering the effects of stochastics and directly incorporating the target number of copies in each of the partitions in case of multiplexed networks. Even though a special case of cTnI has been considered in this study, the model and analysis are analyte agnostic and may be applied to a wide class of chromatographic immunoassays. We believe that this contribution will lead to more efficient designing of the immunochromatographic assays.

## Introduction

**Figure 1:**
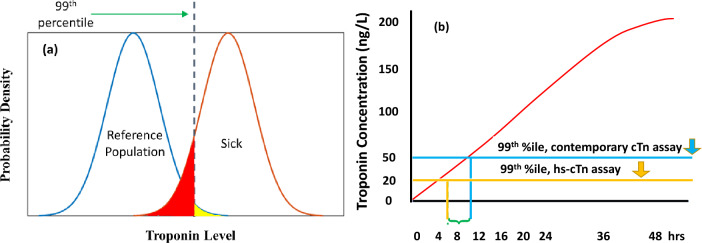
(**a**) When the 99th percentile is the upper limit of the reference interval, the healthy persons in the yellow part of the distribution will get a false-positive result, and the sick persons in the red part of the distribution will get a false-negative result. (**b**) Illustrating that hs-cTn assays (limit set at 20 ng/L) as shown by the yellow line, detect troponin roughly 4–5 h earlier than the conventional assays (limit set at 50 ng/L) as shown by the blue line.

In this contribution we summarize and model how advances in microfluidics can be applied to high sensitivity troponin detection. Troponin is the American College of Cardiology and American Heart Association preferred biomarker for diagnosing acute myocardial infarction (MI)^1^. Troponin T (TnT) and I (TnI) feature amino acid sequences found only in cardiac tissue, making their presence highly specific for cardiac damage^2–4^. A high-sensitivity cardiac troponin (hs-cTn) test refers here to a good analytical limit of detection (LOD), not the clinical sensitivity. In this context, a hs-cTn test means a very low LOD, featuring quantification at levels impossible to achieve with earlier troponin assays. For clinicians, on the other hand, a test’s sensitivity and specificity depends on where a demarcation line between positive and negative test results is drawn. Since increasing sensitivity of a test decreases its specificity, often a demarcation or cut point is drawn at a point that maximizes the sensitivity and specificity of the test. In 1999, an international panel of experts decided on using the 99th percentile of a normal population as a cut point. Heart attack diagnosis is then based on a troponin > 99th percentile of the reference range with a coefficient of variation (CV) of < 10% at that concentration. In the area above the 99th percentile (Fig. 1a), the healthy persons in the yellow part of the distribution will get a false-positive result, and the sick persons in the red part of the distribution will get a false-negative result. In Fig. 1b we illustrate how hs-cTn assays (at 20 ng/L) detect troponin 4–5 h earlier than contemporary assays (at 50 ng/L).

**Figure 2:**
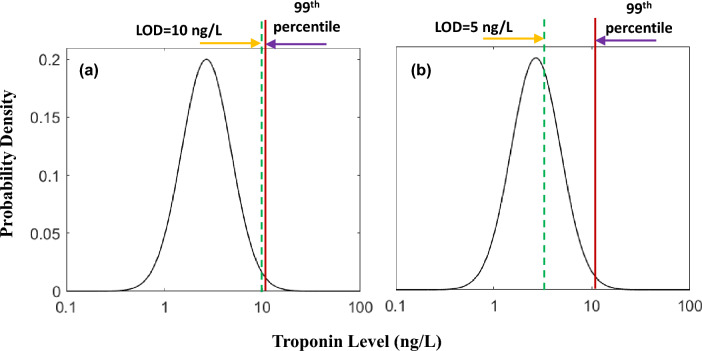
Distribution of troponin results for a normal population for two limits of detection (LODs) indicated by the green dashed lines. (**a**) LOD = 10 ng/L. (**b**) LOD = 5 ng/L. The 99th percentile cut points for both the assays are shown by the solid red lines.

In the modeling work below, we set as an analytical goal an LOD of < 10 ng/L. To defend this goal for yet higher-sensitivity micro-fluidic troponin platforms^5^, we put this in the context of recent medical concerns that newer troponin assays with higher and higher analytical sensitivity will create more and more false positive test results^6^. To this end, in Fig. 2 we illustrate a troponin assay with an LOD1 of 10 ng/L (left panel) and one with an LOD2 of 5 ng/L (right panel). With the lower sensitivity LOD1 assays, troponin is not detected below the 99th percentile cut-point, but with the better LOD2, troponin is detectable well below the 99th percentile cut-point (see the portion of curve to the right of the green dashed line). The 99th percentile cut points for both assays remain the same, since we can safely assume that the shape of the distribution curves for the two assays will be similar. It is thus important to recognize that lowering the LOD for an assay may have no effect on the 99th percentile cut point that determines the upper limit of the normal range if the distribution curves remain the same.

All this means is that one can no longer assume that any detectable troponin is abnormal, and each troponin result needs to be compared to the upper limit of normal for that assay, as one does for the interpretation of other clinical laboratory tests and in this way, these newer assays will not change the false positive rate. Improved analytical sensitivity of the newer assays will enable more rapid and more effective rule-in and rule-out strategies^6,7^.

A widely preferred format of chromatographic immunoassays for developing point-of-care (POC) microfluidic devices for rapid detection of cTnI is in in the form of a lateral flow immunoassay (LFIA)^8^. In its standard format, there is no active control over the flow through the porous matrix of the paper strip. Consequently, this results in a lack of control over the residence time in various zones on the strip, and therefore, an active control over the reaction is lost. This directly leads to poorer LODs. Attempts at offsetting this drawback takes multiple forms like developing better antibodies, signal enhancement techniques, utilizing alternative labels, active mixing, and utilizing alternative porous matrix^9–12^. Each of these attempts leads to an increase in the level of sophistication, and therefore, the cost. In an attempt at achieving the desired LOD of 10 ng/L of cTnI, Choi et al. devised a dual gold nanoparticle (AuNP)-antibody conjugate based LFIA, wherein the first conjugate would bind with the analyte, whereas the second conjugate immobilized downstream, would bind with the first conjugate-antibody complex^13^. Seo et al. developed synthetic antibodies with custom fluorophores to address the issue of quenching of fluorescent molecules to control the molecular distance in a single conjugate unit^14^. This resulted in an LOD < 10 ng/L in a chromatographic immunoassay format. Cai et al.^15^ also attained a high LOD of 16 ng/L by developing red microspheres for enhanced detection.

The attempts to attain high a LOD of cTnI in the above examples clearly show that some complex improvement in one or more of the steps of the assay is necessary. In this work, we show that an alternative arrangement of the capillary immunosensors where the antibodies are immobilized at the reactor wall, can aid hs-cTnI detection by careful designing of the apparatus ^16,17^. This class of immunoassays typically employ immobilization of antibodies on a solid support (capillary tube in this work) as in the case of flow displacement assays and sandwich assays. The analyte then flows over these immobilized agents (either antibodies or antigens, as the case may be) to enable a reaction between the analyte in the solution and the immobilized antibody/antigen at the surface. In case of displacement assays, the non-labeled analyte from the sample displaces the (typically) fluorescently labeled analyte molecules from the immobilized antibody and the collection of these displaced labeled molecules is measured with a fluorescence detector placed at the end of the tube. In the case of sandwich immunoassays, a capture antibody is immobilized at the solid support which binds to the antigen from the solution and then, a detector antibody (also called secondary antibody) binds to the capture antibody-antigen complex^17^.

Irrespective of the type of flow assay described above, there is a critical step of binding of the analyte from the solution that is pumped into the capillary tube, with the immobilized capture antibodies. Some of the important factors that affect this step are the flow rate at which the solution is pumped-in, the diameter and length of the reactor, type of capture antibody, the diffusion of the analyte and the inlet concentration. If the analytes do not bind to the capture antibodies at certain locations of the support, then there will be no displaced labeled analyte from those antibodies (in case of displacement assays) or no subsequent binding of the secondary antibodies at those locations (in case of sandwich assays). Needless to say, this assertion is not affected by the choice of detection scheme.

In this paper, we investigate the influence of the operating parameters over design considerations for a hs-cTnI assay. All the analysis and results are generated for displacement assays, but we note that they are also useful for sandwich assays, as their first step is again, analyte binding with the capture antibody. However, to determine the exact point of exhaustion for a sandwich assay, a secondary binding step needs to be considered in a more complex model of fluid and species transport^16,18^.

The paper is organized as follows: we first provide the analytical model with the governing equations to model the phenomenon along with the typical property values that are relevant for a cTnI assay. Next, we analyse the influence of various control parameters on the assay along with the results for multiplexed assays, specifically in the case of the Simple Plex™ assay^19^. We also provide an updated form of the LOD equation to calculate the minimum necessary volume to detect a particular concentration.

## Analytical model

Modeling of reactions in the course of immunochromatographic assays has received adequate attention for largely the following two configurations: first, lateral flow test kind of arrangements where the antibodies are immobilized in well-defined regions downstream of the sample inlet, and second, bead-based assays where the antibodies are immobilized on beads (small particles) that are present throughout the bulk of the material downstream of the inlet. However, consideration of immobilized antibodies at the wall for flow immunosensor^20^, circumvents the issue of viscous damping during the flow through a porous matrix (in case of lateral flow tests) or the beads. Another difference here is the flow control by an external pumping mechanism (like a syringe pump) that is absent in case of other two arrangements of lateral flow tests and bead-based assays. A commercial example of such an arrangement is the Simple Plex™ assay on Ella instrument^19^.

Troponin-I transport equations are modelled and solved in COMSOL Multiphysics™. The flow is treated to be laminar and incompressible. The fluid transport equation (Navier–Stokes momentum equation) and the species transport equation (advection–diffusion equation) are solved for steady state solutions. The set of simplified governing equations and boundary conditions is provided in Table 1.

**Table 1:** Simplified governing equations and boundary conditions for fluid transport and species transport.

Fluid transport equations	
$\left( {\vec{u} \cdot \nabla } \right)\vec{u} = \frac{\mu }{\rho }\nabla^{2} \vec{u} - \frac{1}{\rho }\nabla p$	Equation (1)
$\frac{\partial }{\partial \theta }\left( {} \right) = 0$	Equation (1a)
$u_{\theta } = 0$	Equation (1b)
$\vec{u}\left( {r = R} \right) = \vec{0}$	Equation (1c)
$\left. {\frac{{\partial \left( {} \right)}}{\partial r}} \right|_{r = 0} = 0$	Equation (1d)
$\frac{{\partial u_{r} }}{\partial z} = 0$	Equation (1e)
$\left. {u_{z} } \right|_{z = 0} = \frac{{\text{Flow Rate}}}{{\text{Cross - sectional Area}}} = u_{in}$	Equation (1f)
$\left. p \right|_{z = L} = p_{atm}$	Equation (1 g)
Species transport equations	
$\vec{u} \cdot \left( {\nabla c} \right) - D\left( {\nabla^{2} c} \right) = 0$	Equation (2)
$\frac{\partial c}{{\partial \theta }} = 0$	Equation (2a)
$\left. {\frac{\partial c}{{\partial r}}} \right|_{r = 0} = 0$	Equation (2b)
$\left. {\left( {\vec{u} \cdot \vec{n}_{z} } \right)} \right|_{z = 0} = u_{in} C_{0}$	Equation (2c)
$D\left. {\frac{{\partial^{2} c}}{{\partial r^{2} }}} \right|_{r = R} = - k\left. c \right|_{r = R}$	Equation (2d)
$D\left. {\frac{{\partial^{2} c}}{{\partial z^{2} }}} \right|_{z = L} = \left. {\left( {u_{z} c} \right)} \right|_{z = L}$	Equation (2e)

**Figure 3:**
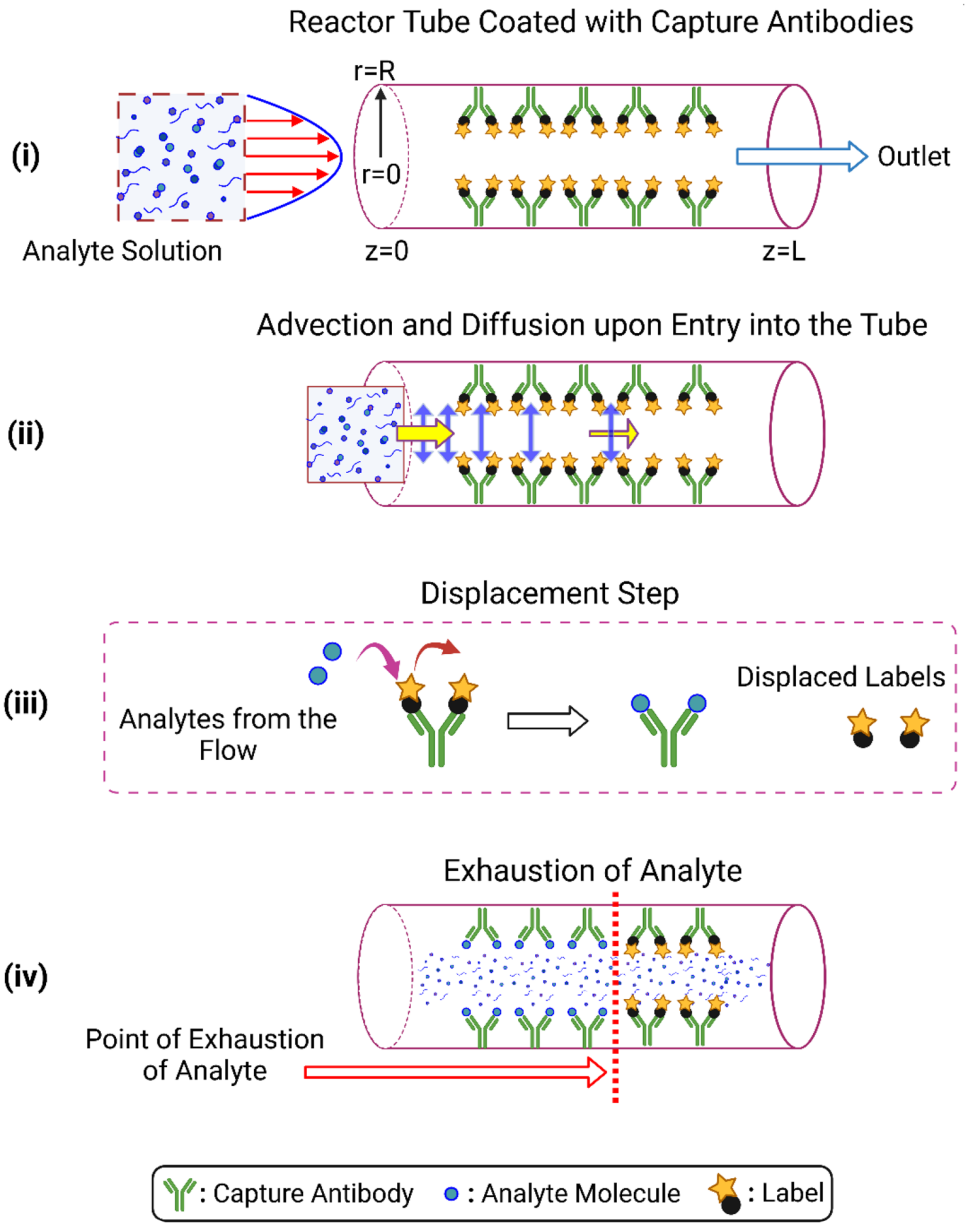
Schematic. (i) The wall of the reactor tube (or capillary tube) is coated with labeled capture antibodies. The analyte solution is pumped into the tube. (ii) Upon entry, there is a two-way transport that takes effect: dominant advection in the streamwise (indicated by yellow horizontal arrow) and diffusion in the cross-stream (indicated by vertical purple arrow) directions. (iii) The analyte molecules displace the fluorescent labels from the sites of the antibodies. (iv) Due to consumption of the analyte at the wall and reduced transport from the middle of the tube, there is a point of exhaustion along the tube length beyond which the concentration of analyte is too low, fixed at 1 aM (10^–18^ M or 23.8 fg/L cTnI) in this study.

The set of equations provided in Table 1 fully describes the system. The symbols $r$, $\theta$, $z$ are the co-ordinates of a cylindrical system, whereas $L$ and $R$ are the length and radius of the reactor, respectively (Fig. 3i). $c$ is the concentration of the analyte, $\vec{u}$ is the velocity of the flow and $u_{r}$, $u_{\theta }$ and $u_{z}$ are the velocity components in the radial, azimuthal (angular) and the axial directions, respectively. $\vec{n}_{z}$ is the unit normal vector along the axis of the tube. $u_{in}$ is the average inlet velocity of the fluid. $C_{0}$ is the initial concentration of the analyte solution that is being pumped into the tube. $D$ is the diffusion coefficient of the analyte and $k$ is the reaction rate constant (Fig. 3ii). Equations (1) and (2) are the governing differential equations for fluid transport and species transport respectively, whereas Eqs. (1a)–(1 g) and Eqs. (2a)–(2e) are the boundary conditions of the corresponding transports. Axial symmetry (Eq. (1d) and (2b)) and azimuthal symmetry (Eq. (1a) and (2a)) is considered in the system. The wall of the reactor is impermeable (Eq. (1c)) and a fully developed flow is considered (Eq. (1f) at the inlet, whereas the outlet is open to atmosphere (Eq. (1 g)). There is a flux of the analyte at the inlet (Eq. (2c)), whereas there is a balance between the diffusion and advection of the analyte at the exit of the reactor (Eq. (2e)). The immunoassay reaction (capture of the analyte by its antibody immobilized at the wall) is modelled by the wall boundary condition as given by Eq. (2d) (Fig. 3iii,iv).

The theoretical model described above is valid for any species transport and we apply it for cTnI transport in this study. Table 2 lists some of the typical property values that are relevant for the present study. We note that these are only representative values and specific property values are provided on each of the graphs in the results and discussion section.

**Table 2:** Typical property values used for immunoassay of cTnI.

Property	Value
Diameter of tube (*Dia*) ^20^	$800 \mathrm{\mu m}$
Length of tube $\left(L\right)$	$10 \mathrm{mm}$
Diffusion coefficient $\left(D\right)$ ^21^	$95\times {10}^{-8} {\mathrm{cm}}^{2}/\mathrm{s}$
Volumetric FLOW RATE (*Q*) ^20^	$0.25 \mathrm{mL}/\mathrm{min}$
Inlet concentration (*C*_*0*_)	$0.42 \mathrm{pM}$ or 1 $0 \mathrm{ng}/\mathrm{L}$
Rate constant for different capture antibodies $\left(k\right)$ ^22^	
19C7	$11.9\times {10}^{5} {\mathrm{M}}^{-1}{\mathrm{s}}^{-1}$
4C2	$6.54\times {10}^{5} {\mathrm{M}}^{-1}{\mathrm{s}}^{-1}$
MF4	$4.95\times {10}^{5} {\mathrm{M}}^{-1}{\mathrm{s}}^{-1}$
8E10	$2.54\times {10}^{5} {\mathrm{M}}^{-1}{\mathrm{s}}^{-1}$

Suitable variations in these values are considered and explicitly stated wherever applicable.

## Results and discussion

An understanding of the influence of various control parameters like the inlet analyte concentration, volumetric flow rate, reactor diameter and choice of antibody is necessary for efficient designing of the immunoassay reactor and/or a network of such reactors^17,23^.

### Axial dispersion of analyte concentration

**Figure 4:**
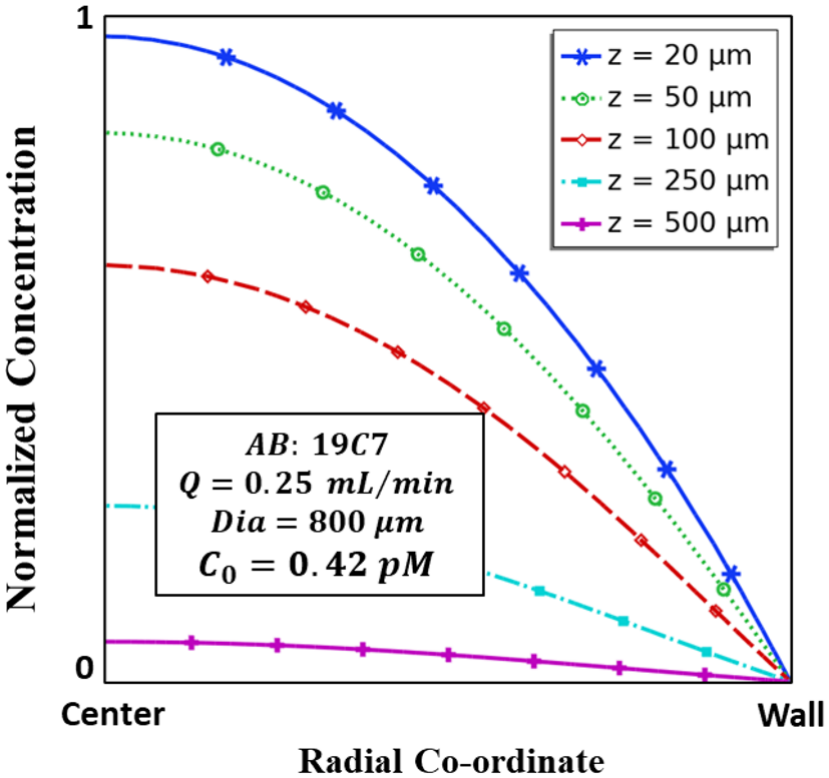
Axisymmetric concentration profile along the length of the tube at various distances (*z*) from the inlet. The property values are listed on the graph. These are the representative values provided in Table 2.

A flattening of the concentration profile is observed along the direction of flow (Fig. 4). This is attributable to the fact that besides the parabolic flow velocity profile, an additional mass transport by diffusion initiates as soon as the analyte enters the reactor. The mass transport thus has both an axial as well as a lateral component. Advection dominates the axial mass transport, whereas diffusion is the sole driving factor in the radial/cross-stream/lateral direction. The radial diffusion is caused by the binding of the analyte to the immobilized capture antibody at the wall. Therefore, there is a gradual spreading out of the species towards the wall during the flow leading to a decay in the dispersion of the concentration profile.

### Influence of reactor control parameters

**Figure 5:**
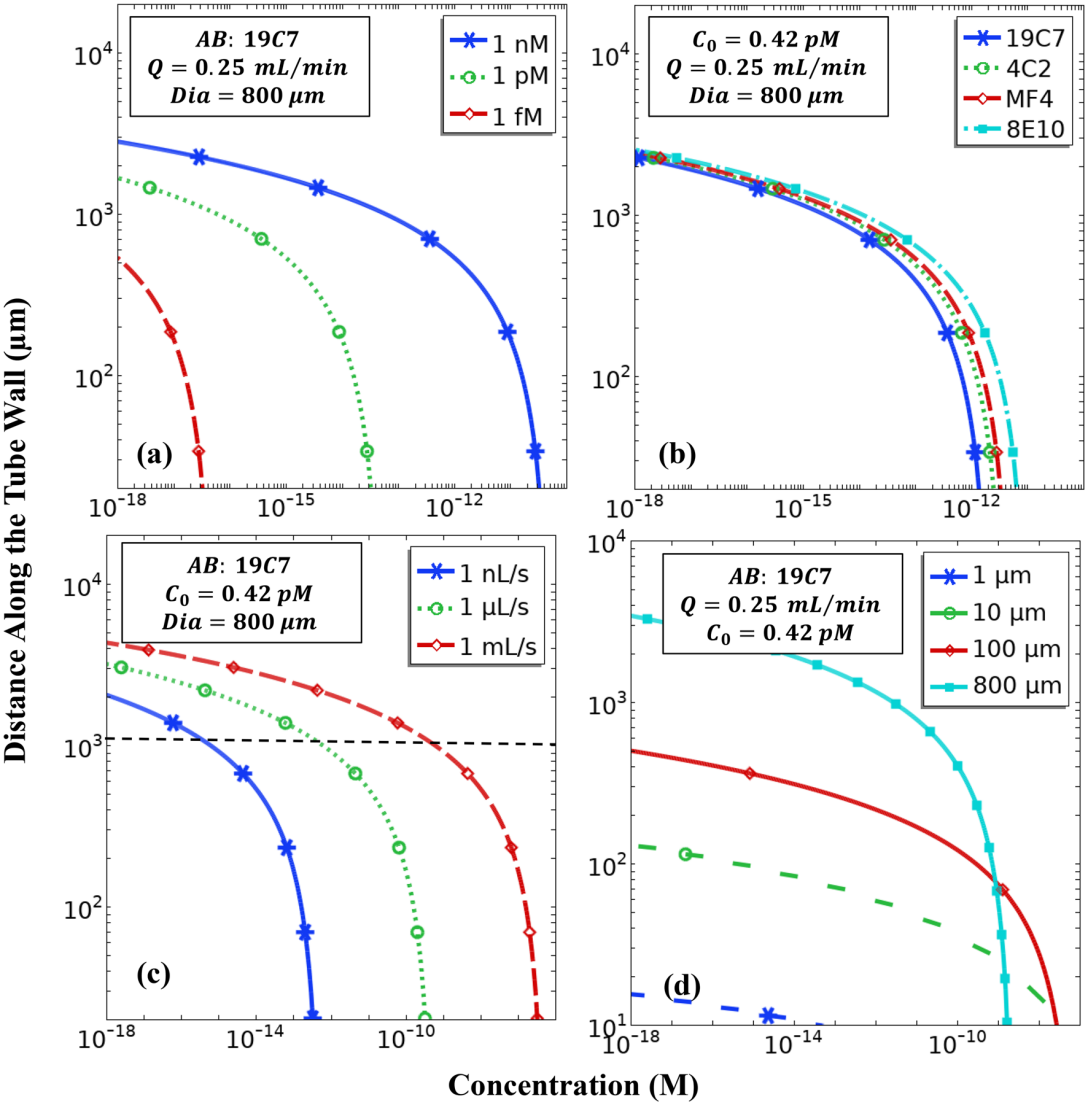
Influence of various control parameters on the required tube length for an immunoassay reactor. The property values are indicated on the respective graphs. Only one parameter is varied at a time for each of the four graphs. In the graphs, ‘*AB*’ refers to Antibody, ‘*Q*’ denotes the volumetric flow rate, ‘*Dia*’ specifies the reactor diameter and ‘${C}_{0}$’ specifies the inlet concentration. Concentration along the tube wall for different (**a**) inlet concentrations of cTnI, ‘${C}_{0}$’, (**b**) capture antibodies placed at the wall of the tube, ‘*AB*’ (**c**) volumetric flow rates, ‘$Q$’ and (**d**) diameter of the tube, ‘$Dia$’.

It is important to find out the location on the tube wall beyond which there is no contribution to the fluorescence signal. This location will be the point on the reactor wall beyond which antigen is depleted. Due to a gradual decay in the nature of the concentration curves (Fig. 5), a concentration of 1 aM (10^–18^ M or 23.8 fg/L) has been chosen as the limiting value, i.e., a concentration smaller than this has been assumed to be too low within the framework of capillary immunosensors. Therefore, whenever 1 aM is reached on the tube wall, it is considered that there is no antigen present beyond this location, and hence, it can be said that all the antigen has been exhausted. The sensing being done here is essentially mass sensing, i.e., the absolute amount of the initial volume affects the output signal. This is discussed further in the section on LOD.

Figure 5a depicts the sensitivity of the assay to the inlet analyte concentration for the indicated property values. It is observed that a higher concentration cTnI solution necessitates longer reactors as compared to lower concentration solutions. Or also, the detector may be placed at shorter distances for a higher sensitivity cTnI assay. An inlet concentration as low as 1 fM (or 23.8 pg/L) can be detected within $0.5 \mathrm{mm}$ or $500 \mathrm{\mu m}$ length of the tube. It is also evident from Fig. 5b that the choice of capture antibodies for cTnI does not have a significant influence over the design considerations of the reactor, and a tube length of $\sim 2\mathrm{ mm}$ suffices for detecting the target concentration of $10\mathrm{ ng}/\mathrm{L}$ (or 0.42 pM). This is because the rate constants are comparable in magnitude (of the same order, see Table 2) and over time, the difference in magnitudes is not large enough to warrant a change in design considerations of the reactor. Since different antibodies have different rate constants, a difference is observed in terms of the absolute concentration at the wall. A clear effect of the volumetric flow rate is visible on the required length (Fig. 5c). A higher flow rate necessitates a longer reactor (albeit slightly). This is explained by the fact that a higher flow rate implies a greater flow velocity which leads to a stronger advection component of the mass transport for the same cross-stream diffusion, whose strength is established by the diffusion coefficient $D$ and the consumption at the reactor wall due to the surface reaction. This directly leads to the species being carried to a longer distance in the streamwise direction for a higher flow rate. Not only this, a higher flow rate also implies a greater amount of analyte being carried into the reactor leading to greater concentrations at the wall. Its direct implication is the effect on LOD. Huang et al.^24^ have recently reported a microfluidic device for hs-cTnI detection based on a sandwich assay whose performance was comparable to a traditional immunochromatographic reagent test strip. They have found a direct dependence of the LOD (pg/mL or M) on the inlet flow rate of the analyte solution. We make the same observation from Fig. 5c. A horizontal line (dashed) at ~ 10^3^ µm intersects the curves as follows: 1 nL/s at ~ 1 fM, 1 µL/s at ~ 1 pM and 1 mL/s at ~ 1 nM. It essentially means that for a reactor of 1 mm length, LOD for a flow rate of 1 nL/s is 1 fM, for 1 µL/s it is 1 pM and for 1 mL/s it is 1 nM. A slower flow facilitates a better LOD as the residence time in the capillary tube is increased and even the lower concentrations of the analyte are transported to the reaction sites, i.e., the reactor wall. A change in diameter of the reactor, however, has a much more prominent effect on the required length of the reactor. (Fig. 5d). It is evident that a smaller diameter tube warrants a shorter tube in comparison to a broader tube, as the analyte is exhausted much earlier. This is attributable to the fact that a narrower tube facilitates a shorter radial distance over which the analyte must be diffused to reach the wall. Hence, a more efficient diffusion to the wall from the middle of the tube leads to a greater availability of the antigen at the wall, and therefore, a greater consumption as well. Not only this, a larger diameter tube accepts a greater amount of analyte, which gets transported over longer distances. A major factor that leads to the observed behaviour is the S/V ratio, i.e., Surface Area/Volume ratio. This scales as the inverse of reactor diameter. For a narrower reactor, S/V is high, implying that a greater surface area is available for the same volume of species and fluid. Since, the capillary immunoassay is essentially a surface reaction, a higher S/V increases the net reaction and hence, the analyte is exhausted at an earlier point on the reactor wall. In essence, choosing a narrower tube leads to lowering the required reactor length. It is clear from the preceding discussion that a 5 mm long reactor suffices to generate a reliable signal for cTnI within the practical framework of considered control parameters.

### Limit of detection (LOD)

In case of multiplexed networks that process a low concentration analyte solution, the statistical distribution of the analyte molecules becomes important, as there could be significant statistical variation in the number of molecules per partition, and some of the partitions may not contain enough molecules to get a detectable signal for the targeted sensitivity^25,26^. Hence, it is necessary to avoid a Poisson distribution to make sure that even in the case of unequal distribution of the sample between the partitions, no partition is likely to be empty. It was shown by Basu et al.^25^ that to avoid a Poisson distribution of the probability to find an analyte molecule in a given volume, $\lambda >3$ must hold, where $\lambda =m/n$ gives the number of copies of the target analyte per volume per partition. This is necessary to avoid the stochastic regime of the concentration vs volume chart and ensure that the chosen volume for a target concentration will contain enough analyte molecules.

**Figure 6:**
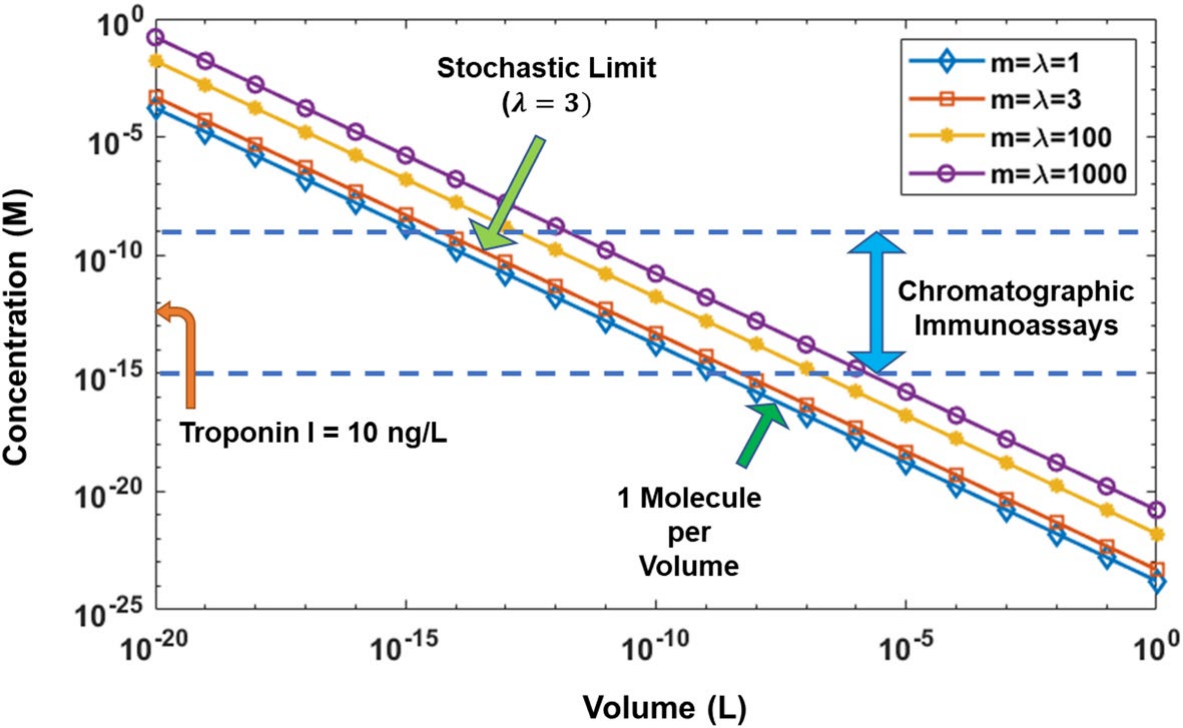
Concentration versus sample volume plot for typical analyte detection/analysis technologies. In the legend, *m* is the target number of copies (or molecules) that is to be detected per sample volume whereas, $\lambda$ is the ratio *m/n* or number of copies per volume/number of partitions, where *n* stands for the number of partitions. This graph is drawn for *n* = *1*. The orange arrow on the concentration axis indicates the concentration level necessary for a LOD = 10 ng/L (0.42 pM) of cTnI. The horizontal region between the two blue dashed lines indicates the typical concentration range (1 nL to 1 fL) that is of interest in fluorescent immunochromatographic assays.

The necessary minimum volume to detect a particular analyte concentration is given as $V = \frac{n \times m}{{\eta N_{A} c_{i} }}$, where $V$ is the total sample volume, ${c}_{i}$ is the concentration of the analyte, ${N}_{A}$ is the Avogadro’s constant $=6.02\times {10}^{23} {\mathrm{mol}}^{-1}$, $\eta$ is the efficiency of the sensor, *m* is the number of copies or molecules of the analyte per volume and *n* is the number of partitions. In Fig. 6, we show the graphs for *n* = *1*. In case of cTnI detection with an LOD of 10 ng/L (or 0.42 pM), the corresponding necessary volume per partition turns out to be ~ 12 pL. We note here that this is for an ideal case of a perfect sensor with 100% efficiency and no inhibitors in the sample solution. However, this is seldom the case. Considering a sample case where sensor efficiency is 50%, the volume turns out to be ~ 24 pL. Now, if we consider 20 partitions in the microfluidic network, this volume becomes 480 pL or ~ 0.5 nL. With practical considerations of inefficient reactions, presence of inhibitors in the analyte solution and confinement effect due to the size of the reactor, this volume could become much larger^27^.

### Multiplexed immunoassay

**Figure 7:**
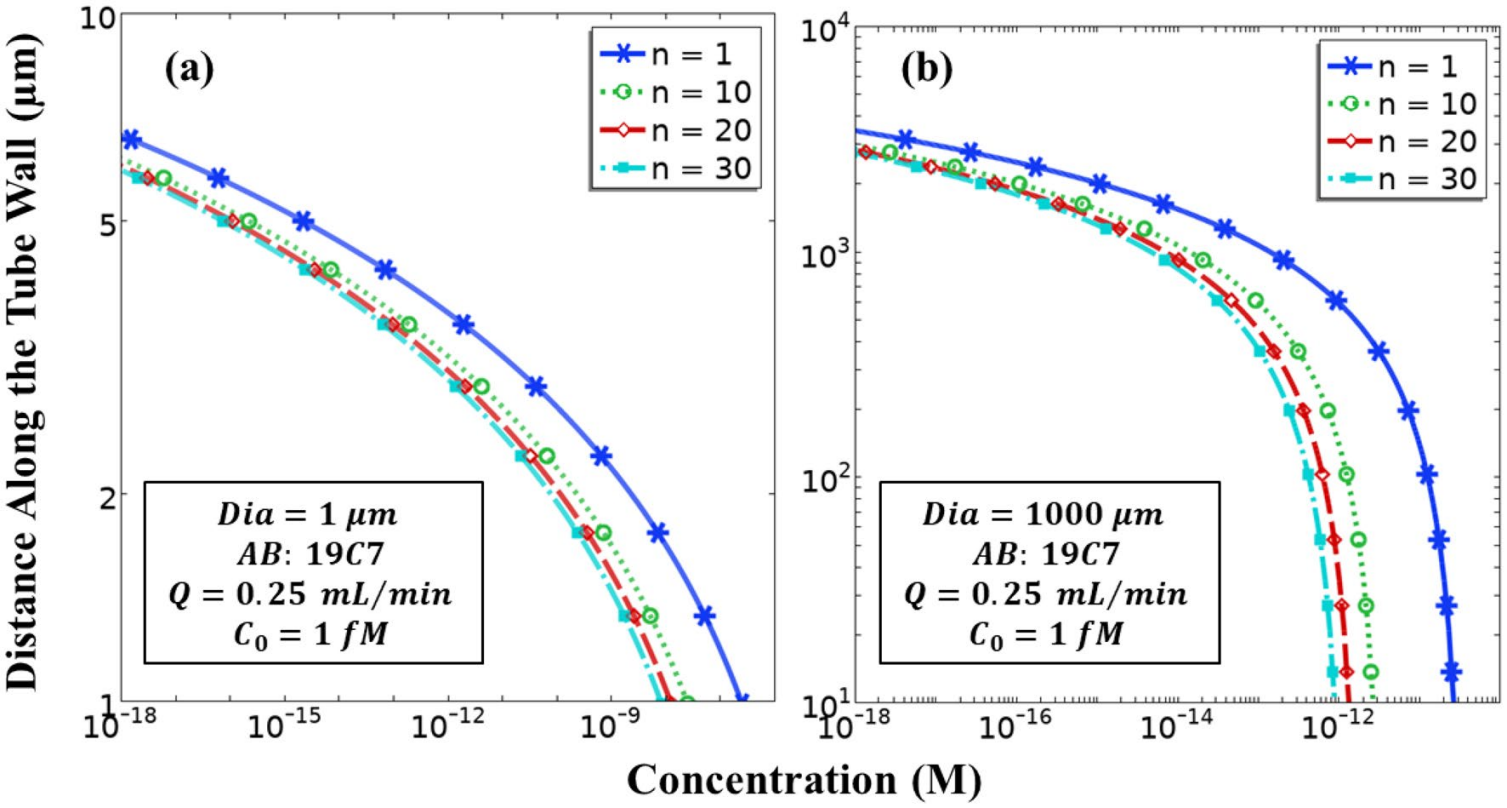
Multiplexed immunoassay. Here *‘n’* denotes the number of parallel channels into which the flow from a common inlet is divided. (**a**) Diameter of each reactor tube is $1 \mathrm{\mu m}$, (**b**) Diameter of each reactor tube is $1000 \mathrm{\mu m}$ or $1 \mathrm{mm}$.

Microfluidics offers the unique possibility to integrate multiple fluidic channels to accomplish a series of objectives in a small space, thereby exerting less pressure on real estate requirements. Here, in Fig. 7, design requirements of an individual reactor are shown when a network of such reactors is utilized for cTnI assay. The property values are indicated on the graphs. The results for the limiting cases of small ($1\mathrm{ \mu m}$) and large ($1000\mathrm{ \mu m}$ or $1\mathrm{ mm}$) diameter reactors are similar in nature. For the same control parameters such as flow rate, inlet concentration and choice of the antibody, reactor of a few microns length is sufficient for a $1\mathrm{ \mu m}$ diameter tube. The observed difference of the required length for different numbers of channels is a few micrometres. However, in case of large diameter reactors (dia ~ $1\mathrm{ mm}$), the difference in the required length is of the order of millimetres and is significant enough to be considered while making the choice of the number of channels in a multiplexed fluidic network. While attempting to incorporate as many channels as possible in a fixed microfluidic cartridge space, saving a few extra millimetres per reactor length could be handy.

A difference is also observed in terms of the concentration profiles along the reactor wall. This directly translates to the strength of the fluorescence signal that will be generated per reactor. Incorporating a larger number of reactors in the multiplexed network will generate a stronger signal if the same analyte is being detected in all the reactors. This will eventually enable the use of fluoresce detectors which may not be able to detect very low signals, as the amplification of the net fluorescence signal itself will allow a detector of relatively low sensitivity to capture the amplified signal. This not only puts lesser constraints on the quality of detectors, but also allows the detection of smaller concentrations of cTnI with the same detector, as the signal is now amplified not due to a greater amount of the analyte, but due to an increase in the number of parallel simultaneous signals.

### Simple Plex assay on Ella

**Figure 8:**
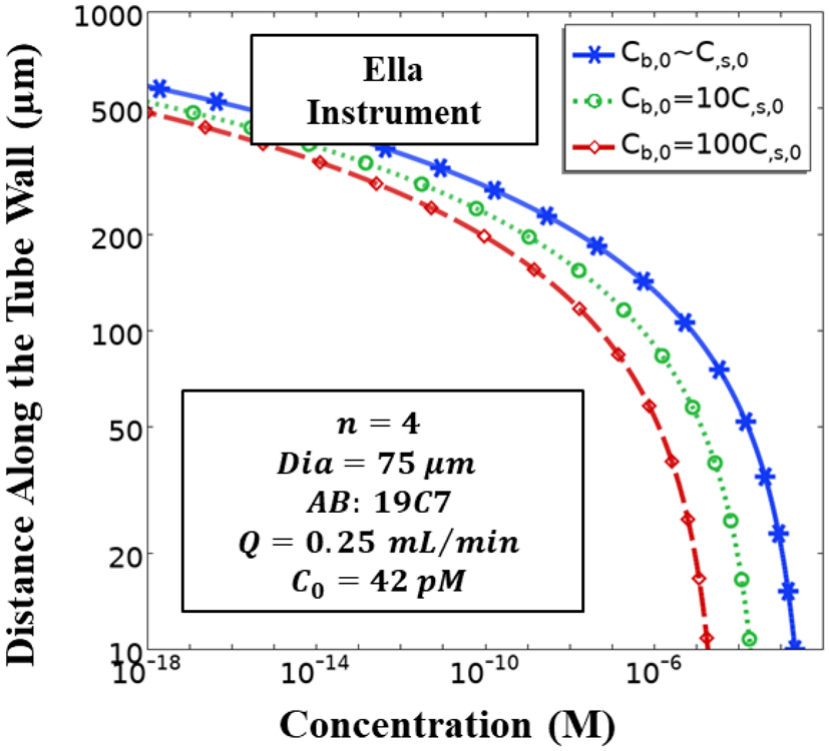
Solution of the Simple Plex™ assay on Ella Instrument. The geometric and material properties are indicated in the box. ‘$\mathrm{n}=4$’ implies that the common inlet flow at $0.25\mathrm{ mL}/\mathrm{min}$ is divided into 4 downstream channels.

A commonly utilized configuration of the multiplexed immunosensor is that of the Simple Plex™ microfluidic platform in Ella Instrument. To summarize, a series of reactors coated with capture antibodies are placed sequentially in a single capillary. Then, there is a network of such capillary tubes (usually 4) which has a common inlet for analyte solution, buffer solution and a common outlet for waste. Depending on the targeted activities, these reactors (a total of 12 reactors upon considering 4 capillary tubes, and 3 reactors per capillary) can be coated with one or more antibodies. These are most suitable for multi-analyte detection simultaneously in a common sample. This could also be used for verifying the signal generated from one capillary tube with the aid of other parallel tubes. At present, Simple Plex™ assay is commercially available in the form of custom assay panels that utilize Simple Plex™ technique on Ella platform, offered by Bio-Techne corporation^28^. Ella is the commercial machine that executes the assay, whereas Simple Plex™ cartridges contain the reactors. The cartridges are available in a range of configurations. The maximum number of parallel tubes is 4, whereas the maximum number of samples that can be processed in a single run is 72. All the cartridge formats are accompanied by pre-loaded calibration curves for a range of assays to choose from. The Ella platform delivers results in less than 90 min. Aldo et al.^19^ have reported a comparison of the performance of Ella instrument with ELISA and Luminex (bead-based assay) type assays and have found good agreement among them. Figure 8 shows that for the typical commercial Simple Plex™ which has GNR (gold nano reactors) tubes of $75\mathrm{ \mu m}$ inner diameter coated with capture antibodies, a length of $500\mathrm{ \mu m}$ per reactor is sufficient to detect $0.42\mathrm{ pM}$ or $10\mathrm{ ng}/\mathrm{L}$ cTnI. Having a tube longer than this will be a waste of resources. This is in excellent agreement with the configuration of Aldo et al.^19^ where the length of each GNR is $250 \mathrm{\mu m}$. Our results show that the design requirements for a much simpler flow displacement assay (in this work) are quite similar to that in case of Simple Plex™ assay on Ella (which has multiple incubation steps in the automated sandwich immunoassay operation). Quite often, microfluidic cartridges require a back-and-forth transport of the analyte solution to ensure that all the analyte is reacted with the antibodies. It is basically another way of implementation of a larger number of capture antibodies at the wall. The results (Fig. 8) show that there is no significant effect of the number of antibodies at the wall over the design parameters. However, a higher density of antibodies at the wall leads to a faster consumption of the analyte, and hence, a decrease in the number of flow passes needed to ensure full consumption of the analyte.

## Conclusions

We have provided design analysis for chromatographic immunoassays (displacement mode) with a special focus on hs-cTnI detection, and an upgrade on the existing LOD equation to take into account the stochastic effects to calculate necessary minimum volume of the sample, for that LOD. The analysis and model are sufficiently generic to be applicable for any single species immunoassay. A major outcome envisaged from this study is establishing the fact that the design requirements of a flow immunosensor are significantly affected by the choice of operating parameters, and also the fact that there exists a critical length of the reactor beyond which the analyte will be depleted. Importantly, this length is greatly influenced by the choice of operating parameters, or conversely, one or more of the operating parameters can be selected to suit the demands of the eventual device where such an assay is to be implemented. This will have a direct implication on more efficient integration of such capillary reactors in microfluidic devices, which will in turn lead to optimal utilization of often limited real estate in such microfluidic devices, especially when the intended use is at POC. Not only this, we have also explained the multiplexed arrangement (with a special case of Simple Plex™ assay on Ella) wherein we show that upon increasing the number of parallel channels in the microfluidic network, the incremental reduction of the required length of each of the reactors is quite low. Even though the choice of analyte here is cardiac troponin I, the fundamental nature of the model governing transports of flow and species allows direct application of this model to any other analyte for a similar assay. Only the analyte specific information like diffusion coefficient and reaction rate constant will have to be changed in the boundary conditions. Other parameters like volumetric flow rate and reactor size (diameter and length) will be dynamic and will depend on the targeted level of sensitivity.

## Data Availability

Data will be made available by the corresponding author upon suitable request.
